# Mutational and non mutational adaptation of *Salmonella enterica* to the gall bladder

**DOI:** 10.1038/s41598-019-41600-8

**Published:** 2019-03-26

**Authors:** Verónica Urdaneta, Sara B. Hernández, Josep Casadesús

**Affiliations:** 1 Departamento de Genética, Facultad de Biología, Universidad de Sevilla, Apartado 1095, 41080 Sevilla, Spain; 2 Present Address: Umeå Centre for Microbial Research, Umeå University, Umeå, 90187 Sweden

## Abstract

During systemic infection of susceptible hosts, *Salmonella enterica* colonizes the gall bladder, which contains lethal concentrations of bile salts. Recovery of *Salmonella* cells from the gall bladder of infected mice yields two types of isolates: (i) bile-resistant mutants; (ii) isolates that survive lethal selection without mutation. Bile-resistant mutants are recovered at frequencies high enough to suggest that increased mutation rates may occur in the gall bladder, thus providing a tentative example of stress-induced mutation in a natural environment. However, most bile-resistant mutants characterized in this study show defects in traits that are relevant for *Salmonella* colonization of the animal host. Mutation may thus permit short-term adaptation to the gall bladder at the expense of losing fitness for transmission to new hosts. In contrast, non mutational adaptation may have evolved as a fitness-preserving strategy. Failure of RpoS^−^ mutants to colonize the gall bladder supports the involvement of the general stress response in non mutational adaptation.

## Introduction

Variation of mutation rates in response to environmental factors is an old prediction of population genetics^1^, and has been confirmed by studies in bacterial populations^2–6^. Different kinds of stress induce different types of mutations, thus supporting the view that increase of the mutation rate under stress is an adaptive response indeed^7^.

A drawback of mutational adaptation is that mutations are often deleterious, and their accumulation in a population can decrease fitness^8,9^. In fact, bacterial populations with high mutation rates adapt quickly to a given environment but are outcompeted by non mutators in the long run^10^. This loss of long term fitness may contribute to explain the evolutionary emergence of non mutational strategies that adapt bacterial populations to cope with environmental challenges^11–13^.

Here, we have investigated the contribution of mutational and non mutational adaptation to bacterial survival in a natural environment, the gall bladder of the house mouse, *Mus musculus* L. The gall bladder is an infamous environment for bacteria due to the presence of high concentrations of bile salts that cause membrane disruption, protein denaturation, and DNA damage^14,15^. However, certain bacterial pathogens are able to colonize the gall bladder, and a relevant example is *Salmonella enterica*^14^. Invasion of the gall bladder epithelium and formation of biofilms can protect *Salmonella* from bile; however, planktonic *Salmonella* cells are also found in the gall bladder lumen^15–17^. Studies *in vitro* have shown that *S*. *enterica* can survive in the presence of a lethal concentration of bile salts by both mutational and non mutational mechanisms^18^. Among the mutations found to confer bile resistance, one class affects lipopolysaccharide transport genes^18^, an observation consistent with the role played by the LPS as an envelope barrier to bile salt uptake^19^. In turn, non mutational adaptation *in vitro* has been shown to involve upregulation of the RpoS-dependent general stress response^18^. Additional stress responses may be also involved, including activation of efflux systems and remodeling of the cell envelope^18,20,21^.

Our investigation of adaptation of *S*. *enterica* to the gall bladder offers the obvious advantage that the setting is a natural environment. A disadvantage, however, is that statistical analysis is hampered by the ignorance of bacterial population sizes inside the gall bladder, of bacterial cell death rates, and of other parameters. Despite this limitation, we present *bona fide* evidence that high mutation rates may occur in the gall bladder, thus suggesting the occurrence of stress-induced mutation. However, a significant fraction of mutations that permit survival in the gall bladder appear to impair other virulence-related traits. We also show that non mutational adaptation to bile is a common phenomenon, as previously observed in laboratory trials^18^. Furthermore, we tentatively propose that the RpoS-dependent general stress response may play a crucial role in gall bladder colonization.

## Results

### Recovery of *Salmonella enterica* populations from murine gall bladders

*Salmonella* infection was performed on two strains of mice: BALB/c, an immunodeficient breed suitable to monitor systemic, acute infection^22–24^ and 129S2/SvPasCrl, an immunoproficient breed that permits monitoring of long lasting, persistent infection^25^. Both types of mice were infected by the oral route.

Two days after oral infection, feces were homogenized in LB to obtain a final concentration of 1 g/l, and aliquots were spread on LB with ox bile. Bile-resistant colonies were not detected, indicating that bile-resistant mutants were rare or absent in the intestine of infected animals.

The nineteen BALB/c mice showed customary disease symptoms after five days, and were euthanized. Gall bladders were recovered, homogenized, and serial dilutions (10^−1^, 10^−2^, 10^−3^, and 10^−4^) were prepared in 0.9% NaCl. To select *Salmonella* isolates avoiding growth of other intestinal bacteria, the dilutions were plated on LB agar with streptomycin and incubated overnight at 37 °C (strain SL1344 is intrinsically resistant to streptomycin). The following day, the colonies were counted. Table 1 shows the total number of colony-forming-units (CFU) recovered from the gall bladder of each BALB/c mouse (applying dilution factors to the calculations when necessary). The same table shows the number of CFU detected upon replica-printing onto plates containing increasing concentrations of ox bile (12–20%). Because the MIC of ox bile for the wild type is 12%, only isolates with stable bile resistance (in other words, mutants) can grow on higher concentrations of bile. Substraction of the number of mutants from the total number of CFU provides the number of isolates that had undergone non mutational adaptation.

**Table 1:** Number of *Salmonella* CFU recovered from the gall bladder of BALB/c mice.

Mouse	Total number of CFU recovered from the gall bladder	Number of bile-resistant CFU after non selective growth
1	63	2
2	548	8
3	105	1
4	475	0
5	6,694	1
6	51	0
7	31,204*	1
8	37	0
9	1,042	1
10	222	40
11	2,858	50
12	203,400*	32
13	182	11
14	422	16
15	202,500*	49
16	1,599,000*	122
17	1,018	20
18	2,783	17
19	11,203*	29

*Inferred from dilution and plate counts.

To analyze the *S*. *enterica* populations that colonized the gall bladder upon long lasting infection, twenty-five 129S2/SvPasCrl mice were infected. Because the mice did not show clinical disease symptoms after infection, mouse feces were periodically collected to confirm ongoing infection^26^. These surveys were performed weekly during the first month, and two-weekly afterwards. The presence of the inoculated *Salmonella* strain was confirmed by observing colony formation on LB agar containing streptomycin. Plating of culture aliquots to LB agar containing 13% ox bile failed to detect bile-resistant mutants in feces. Seven 129S2/SvPasCrl mice cleared the *Salmonella* infection during the first four weeks, and four additional mice during the second month after infection. The remaining fourteen 129S2/SvPasCrl mice were euthanized after 10 weeks of infection. Gall bladders were recovered and homogenized, and *Salmonella* CFU were recovered. Discrimination between bile-resistant mutants and unstable bile-resistant isolates was performed as above. Table 2 shows the total number of CFU and the number of stable bile-resistant CFU present in the gall bladder of each 129S2/SvPasCrl mouse.

**Table 2:** Numbers of *Salmonella* CFU recovered from the gall bladder of 129S2/SvPasCrl mice.

Mouse	Total number of CFU recovered from the gall bladder	Number of bile-resistant CFU after non selective growth
1	556	8
2	14	0
3	5	0
4	78	3
5	23	0
6	8	1
7	27	0
8	720	4
9	45	0
10	236	10
11	231	1
12	49	0
13	89	4
14	16	0

Relevant observations from these trials were as follows:

(i) The numbers of CFU found in the gall bladder showed high variability from one mouse to another (Tables 1 and 2). Note that all mice from each strain (BALB/c or 129S2/SvPasCrl) were genetically identical and that inoculation was performed with identical numbers of cells. High variability suggests that gall bladder colonization may be under the influence of multiple factors, probably with stochastic components including clonal expansion.
(ii) The numbers of *Salmonella* colony-forming-units found in the gall bladders of 129S2/SvPasCrl mice were lower than in BALB/c mice. Furthermore, gall bladders that did not appear to contain *Salmonella* cells were detected in 129S2/SvPasCrl mice but not in BALB/c mice (Tables 1 and 2). Clearance of infection was therefore significant in immunocompetent mice, and absent in immunodeficient mice.

A relevant conclusion from the data shown in Tables 1 and 2 is that the majority of isolates recovered from mice gall bladders appear to derive from *Salmonella* cells that had survived in the gall bladder without mutation. Bile-resistant mutants were not even obtained in certain cases (BALB/c mice #4, #6 and #8, and 129S2/SvPasCrl mice #2, #3, #5, #7, #9, #12, and #14). These observations are in agreement with a previous study of bile resistance *in vitro* describing that non-mutational adaptation to bile was more frequent than mutation^18^. However, the number of bile-resistant mutants was remarkably high. An extreme case was BALB/c mouse #10, which yielded 40 bile-resistant mutants vs 182 isolates with unstable bile resistance. Mutant frequencies cannot be calculated due to our ignorance of critical numbers: the bacterial population size, the number of cell divisions within the gall bladder, the bacterial death rate, etc. Furthermore, bile-resistant isolates from the same gall bladder may be siblings. However, the relative abundance of bile-resistant mutants may suggest the occurrence of adaptive mutations in response to the antimicrobial and mutagenic effects of bile^27,28^.

### Whole-genome sequencing in bile-resistant mutants isolated from gall bladders

Ten bile-resistant mutants of independent origin (each isolated from a different BALB/c mouse gall bladder) where chosen for further study. The bile resistant phenotype of the mutants was confirmed by determination of the minimal inhibitory concentration of sodium deoxycholate (DOC) (Table 3). Full genome sequencing was then performed, including the genome of the laboratory stock of *S*. *enterica* SL1344 as a control. DNA sequencing data were validated by sequencing PCR products generated with high fidelity DNA polymerase. The oligonucleotides used to amplify appropriate DNA regions from each mutant are listed in Table S1. Alignment with the *S*. *enterica* SL1344 genome sequence (ASM21085v2) was used to identify DNA sequence differences. The alignment tool used was the BWA-MEM algorithm^29,30^. Relevant observations were as follows (see also Table 3):

(i) Mutant #1 harbored a transversion in *rlpB*, a gene that encodes a lipoprotein B precursor in *E*. *coli* and is involved in LPS transport^31^. A point mutation in *rlpB* had been previously described in a *S*. *enterica* bile-resistant mutant isolated *in vitro*^18^. Interestingly, *rlpB* has been reported to be upregulated by bile in *S*. *enterica*^32^.
(ii) Mutant #2 harbored a transition in *pbgA*, which encodes a transmembrane protein involved in transport of acidic diphosphatidylglycerols (also known as cardiolipins) to the outer membrane^33^. Transcription of *pbgA* is upregulated by bile^32^.
(iii) Mutant #3 harbored a transversion in the *dipZ* gene. In *E*. *coli*, this gene encodes a protein involved in the formation of disulfide bonds in proteins that are translocated across the cytoplasmic membrane^34,35^. Evidence exists that DipZ may play a role in copper tolerance^36^.
(iv) Mutants #4 and #7 harbor mutations in genes that encode cell division factors (*ftsQ* and *ftsK*) and show increased expression in the presence of bile^32^. In mutant #4, a transversion changes the *ftsQ* gene start codon from ATG to TTG, which is the second most used start codon in *E*. *coli*^37^. Mutant #7 presents an in-frame deletion of 36 nt that results in loss of 12 amino acids. Studies in *E*. *coli* have shown that both FtsQ and FtsK are essential membrane proteins that are recruited to the Z ring and participate in septum formation^38,39^. Interestingly, *E*. *coli* FtsQ has been shown to interact with DamX, a septal protein involved in bile resistance in both *Salmonella* and *E*. *coli*^40,41^.
(v) Four independent mutants (#5, #7, #9 and #10) harbored mutations of various types in intergenic regions. Interestingly, in mutants #5 and #10 the mutations are located in genome regions that are highly induced by bile and include the small RNAs STnc400 and GlmZ^32^.
(vi) Mutant #6 shows a nucleotide substitution in the DNA adenine methylase gene, *dam*. This result was unexpected and difficult to interpret because *dam* mutations usually cause bile sensitivity^27,42–44^.
(vii) Mutant #7 presents mutations in two unknown loci, *STM1268* and *ygcF*. A homolog of *ygcF* has been shown to be activated by copper shock in the plant pathogen *Erwinia amylovora*, and its deletion renders a copper-sensitive phenotype^45^.
(viii) Mutant #8 harbors a transition in the *yhbG* gene (also known as *lptB*, lipopolysaccharide transport protein). In *E*. *coli*, this gene encodes an ABC transporter that may play a role in envelope integrity^46–49^. The *S*. *enterica yhbG* gene was found to be upregulated in a pig model of *S*. *enterica* persistence^50^. Interestingly, alteration of LPS transport in *S*. *enterica* has been previously shown to play a role in bile resistance^18^.

**Table 3:** Mutations present in the genome of bile resistant derivatives of *S*. *enterica* SL1344 isolated from gall bladders.

Mutant #	MIC of DOC (%)^a^	Genome position	Locus affected	Mutation	Predicted change	Cellular function affected
1	14	708234	*rlpB*	G → T	Ala → Glu	Lipopolysaccharide transport
2	13	2326694	*pbgA*	A → T	His → Leu	Outer membrane maintenance
3	13	4589340	*dipZ*	C → A	Ala → Ser	Copper tolerance and cytochrome c biogenesis
4	13	153536	*ftsQ*	A → T	Met → Leu (start codon change)	Cell division
5	14	1072585	Intergenic region STnc400 (sRNA) and STM3845 (hypothetical protein)	Deletion of 1 nt (A)	Unknown	Unknown
6	14	3660275	*dam*	T → A	Synonym amino acid change	DNA methylation
7	14	994451	*ftsK*	Deletion of 36 nt	Loss of 12 amino acids	Cell division
		1306516	STM1268	C → T	Ala → Val	Unknown
		3118294	*ygcF*	A → C	Val → Gly	Unknown
		3216955	Intergenic region STM3034 (hypothetical virulence protein) and STM3036 (hypothetical protein)	G → A	Unknown	Unknown
8	14	3505277	*yhbG* (*lptB*)	G → A	Ala → Thr	Lipopolysaccharide transport
9	13	2728613	Intergenic region *gogB* (effector protein) and STM2585 (hypothetical transposase)	Insertion of 6 nt	Unknown	Unknown
10	14	4162532	Intergenic region *yifK* (probable amino acid permease) and GlmZ (sRNA)	Deletion of 15 nt	Unknown	Unknown

^a^The MIC of DOC for the wild type is 7%.

### Growth of bile-resistant mutants

**Figure 1:**
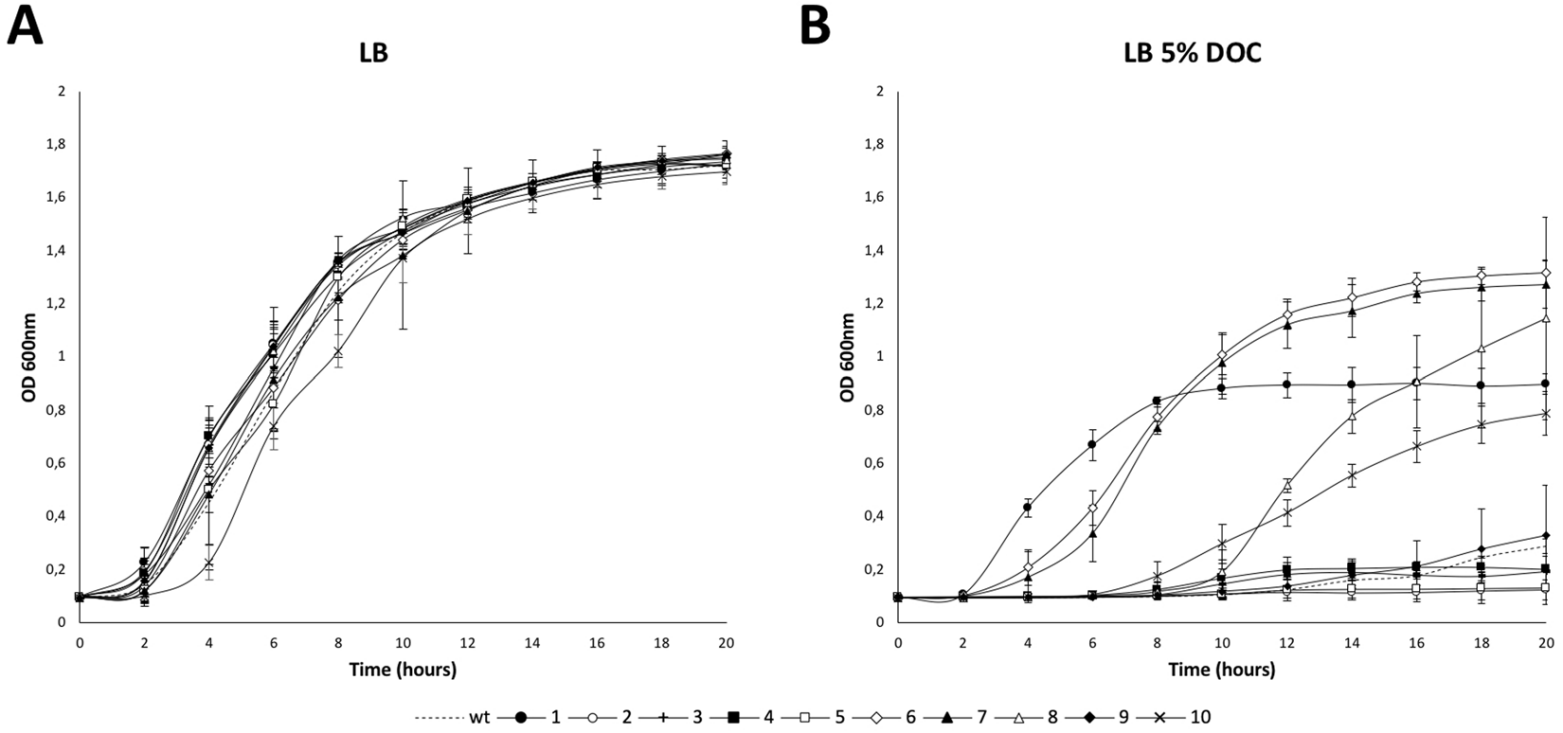
Growth of bile-resistant mutants. Growth curves of *S*. *enterica* bile-resistant mutants isolated from murine gall bladders in LB (**A**) and LB with 5% sodium deoxycholate (**B**). The wild type is included as a control in both graphs. Error bars represent the standard error of the mean from 4 independent replicates.

Because slow growth of certain mutants was observed during MIC determinations, we monitored growth of the 10 bile-resistant mutants under study in LB broth and LB broth supplemented with a sublethal concentration of DOC, including the wild type as control. While the 10 mutants under study grew normally in LB, half of the mutants (#1, #6, # 7, #8 and #10) showed faster growth than the wild type in the presence of DOC, four of the mutants showed slower growth (#2, #3, #4 and #5), and only one mutant (#9) showed a growth rate similar to the wild type (Fig. 1). Growth restraint in specific host niches has been shown to be an adaptive response of bacterial pathogens including *Salmonella*^51–54^. Hence, the possibility that slow growth may contribute to bile resistance may be considered. Albeit speculative, this hypothesis is supported by the observation that non-dividing cultures of wild type *S*. *enterica* show increased resistance to bile salts^55^.

### Analysis of virulence-related traits in bile-resistant mutants

**Figure 2:**
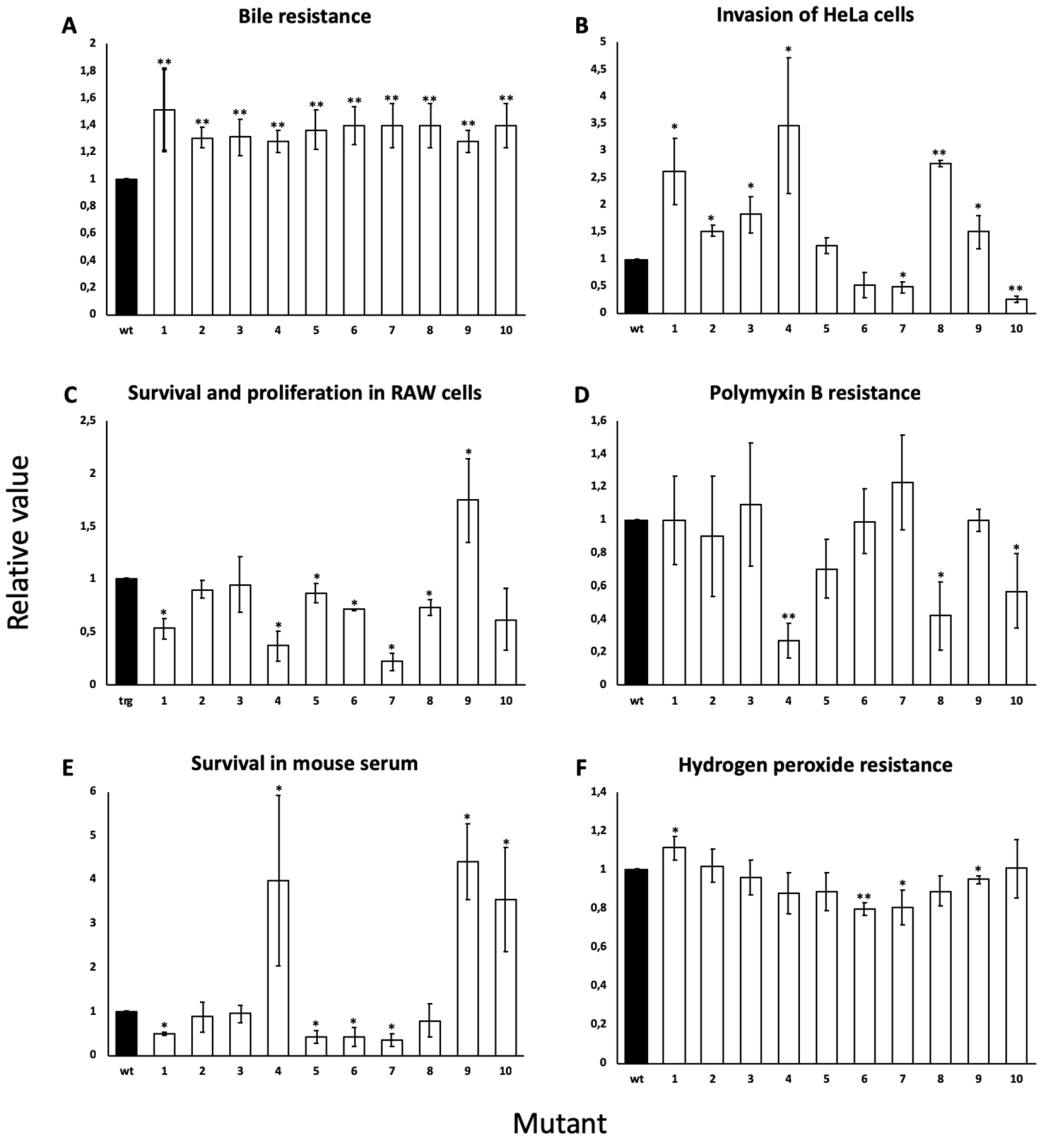
Assessment of virulence-related fitness. Resistance to bile (**A**), invasion of Hela epithelial cells (**B**), survival and proliferation inside macrophages (**C**), resistance to polymyxin B (**D**), survival in mouse serum (**E**) and resistance to hydrogen peroxide (**E**) of the bile-resistant mutants under study. The values obtained for each mutant and condition were normalized to those of the wild type (which was set as 1 for each condition). Absolute chemical concentrations were: DOC, 7%; polymixin B, 0.4 μg/ml; hydrogen peroxide, 0.002%. Standard deviations of 3 independent experiments are shown. Bars with asterisks are significantly different according to the two-tailed *t* test (*P < 0.05; **P < 0.01).

A potential drawback of mutation as an adaptive strategy is impairment of fitness upon environmental change^56^. Because the bile-resistance mutations identified in this study mapped in loci potentially involved in multiple physiological activities, we examined whether they affected virulence-related traits other than bile resistance. For this purpose, we examined the ability of the 10 mutants under study to invade HeLa epithelial cells, to survive and proliferate inside macrophages *in vitro*, to resist polymyxin B (a cationic antimicrobial peptide that interacts with the cellular envelope and is similar to those found in the intestine epithelium), to survive exposure to mouse serum (a test that provides information about the ability of the pathogen to survive the bactericidal activity of the complement), and to resist hydrogen peroxide, an antibacterial molecule produced inside phagocytes. As a control, resistance to ox bile was also tested (Fig. 2A). Relevant observations can be summarized as follows:

(i) Six out of 10 mutants were able to invade epithelial cells more efficiently than the wild type (#1, #2, #3, #4, #8 and #9). Two mutants invaded less efficiently (#7 and #10), and another two mutants (#5 and #6) invaded like the wild type (Fig. 2B).
(ii) Survival and/or proliferation inside macrophages were found to be impaired in 5/10 mutants (#1, #4, #6, #7 and #8). Four mutants (#2, #3, #5 and #10) behaved similar to the wild type, and mutant #9 showed increased survival (Fig. 2C).
(iii) Seven mutants (#1, #2, #3, #6, #7 and #9) showed levels of polymyxin resistance similar to that of the wild type; the remaining mutants (#4, #5, #8, and #10) showed lower levels of resistance (Fig. 2D)
(iv) Survival to mouse serum was found to be lower in 4 mutants (#1, #5, #6, and #7). Three mutants (#4, #9, and #10) showed higher survival and another three (#2, #3, and #8) behaved like the wild type (Fig. 2E).
(v) Wild type (or near-wild type) levels of peroxide resistance were found in most mutants. Exceptions were #1 and #6 (Fig. 2F).

A noteworthy observation is that 7/10 bile-resistant mutants (#1, #4, #5, #6, #7, #8, and #10) showed one or more virulence-related defects. In such cases, one may tentatively conclude that resistance to bile is achieved at the expense of virulence impairment. On the other hand, it is conceivable that increased invasiveness of epithelial cells, especially in mutants #1, #4, and #8 might contribute to gall bladder colonization^16,17^. However, these mutants might be anyway avirulent as they are deficient in one or more virulence traits (Fig. 2). Altogether, the above observations suggest that mutational adaptation to the gall bladder may involve fitness tradeoffs in certain cases. Albeit speculative, this view is in agreement with the evidence that within-host evolution of pathogens can result in loss of fitness for transmission^9^.

### Role of the RpoS general stress response in non mutational adaptation to bile

A previous study *in vivo* provided evidence that activation of the RpoS-dependent general stress response is a major mechanism for non mutational adaptation to bile^18^. Based on this antecedent, we tested the ability of an RpoS^−^ strain to colonize the gall bladder. For this purpose, we co-infected five BALB/c mice with two strains, SV5561 (RpoS^−^) and SV4898 (wild type). Infection was performed by the oral route, and mice were euthanized after 7–10 days. Gall bladders were then recovered, and bacterial cells were plated onto LB agar + kanamycin (to distinguish the wild type) and LB agar + ampicillin (to distinguish the RpoS^−^ strain). Km^r^ and Ap^r^ colonies were then replica-printed to LB + ox bile to determine whether bile-resistant mutants were present. The RpoS^−^ strain largely failed to colonize the gall bladder (Table 4), and bile-resistant mutants were a significant fraction of the rare RpoS^−^ isolates recovered. Failure of the RpoS^−^ mutant to colonize the gall bladder is in agreement with the inefficient colonization of other organs by RpoS^−^ strains^57,58^ and with the sensitivity of RpoS^−^ mutants to bile^18,21^.

**Table 4:** Numbers of CFU recovered from gall bladders of BALB/c mice co-infected with RpoS^+^ and RpoS^−^ strains.

Mouse	Number of RpoS^+^ (Km^r^) CFU recovered from the gall bladder		Number of RpoS^−^ (Ap^r^) CFU recovered from the gall bladder	
	Bile-sensitive	Bile-resistant	Bile-sensitive	Bile-resistant
1	203	7	0	1
2	1,788	141	5	6
3	4,661	194	14	21
4	1,507	11	7	12
5	55,000*	1,106	9	13

*Inferred from dilution and plate counts.

## Discussion

Recovery of *Salmonella* isolates from murine gall bladders yields bile-resistant mutants and isolates that lose bile resistance after non selective growth. Their absolute and relative numbers fluctuate widely, suggesting that colonization of the gall bladder may involve multiple factors, perhaps including stochastic components. This view is supported by a previous study *in vitro* indicating that *S*. *enterica* cultures contain cells that are preadapted to survive bile by two types of stochastic mechanisms: mutation and “noisy” activation of stress responses^18^.

Bile-resistant mutants were recovered at high frequency from gall bladders (Tables 1 and 2). Unfortunately, several hurdles impede the estimation of mutation rates: ignorance of the size of the *Salmonella* populations that had colonized the gall bladder, of the number of cell divisions undergone within the gall bladder, and of the rate of cell death. However, if the populations that colonize the gall bladders of mice are made of less than 50 bacterial cells as shown in other organs^59^, the frequency of mutants may be considered extremely high. The possibility that the bile-resistant isolates recovered from the gall bladder derived from spontaneous mutants present in the inoculum seemed unlikely as bile-resistant mutants were not detected in fecal samples. Occurrence of stress-induced mutation in the gall bladder may thus be tentatively proposed. This tentative conclusion is supported by a previous study showing the occurrence of mutations that improved *Salmonella* colonization of the intestine, and potentially of the liver and the spleen^60^.

The nature of the bile resistance mutations found (Table 3) permits tentative interpretation of their consequences. Most mutations (7 out of 13) map in loci that encode envelope-related structures (*rlpB*, *pbgA*, *dipZ*, *yhbG* [*lptB*], *ftsQ*, *ftsK*, and the intergenic region between *yifK* and *glmZ*), in agreement with previous studies that underline the relevance of the bacterial envelope as a barrier to bile^28,61–65^. Two such mutations (*rlpB* and *yhbG*) map in genes involved in LPS transport, previously shown to play a role in bile resistance^18^. Curiously enough, a mutation in *rlpB* was also found to confer bile resistance in a study that selected bile resistant-mutants *in vitro*^18^. In turn, the discovery of mutations in cell division factors is in agreement with the upregulation of the cell division factor gene *zapB* by bile^18^ and with the occurrence of peptidoglycan remodeling in the presence of bile^66^. A side but interesting observation is that a significant fraction of the loci affected (*yhbG*, *pbgA*, *ftsQ*, *ftsK*, *STnc400*, *rlpB*, and *glmZ*) are known to be upregulated by bile^32^.

Because a fraction of bile resistant mutants under study grew slowly during MIC tests, their growth patterns were monitored. These surveys were prompted by the well known fact that certain bacterial pathogens reduce or arrest growth in host environments, a strategy often known as “dormancy”^67^. In *Salmonella*, dormant-like states have been shown to occur inside phagocytes^51^ and fibroblasts^52,53^. While all the mutants under study showed similar or identical growth rates in LB (Fig. 1), 4 out of 10 mutants showed growth rate reduction in the presence of a sublethal concentration of sodium deoxycholate. Slow growth rate is in sharp contrast with their ability to survive in the presence of a lethal concentration of DOC in MIC assays (>12% in all cases), and may support the speculation that a dormant-like state contributes to bile resistance in certain cases.

A notion well established in the literature is that mutations that confer rapid adaptation in a given environment may not necessarily be beneficial in the long term^9^. In the case of *Salmonella*, which thrives in multiple environments inside and outside hosts, shedding of a virulent population to the environment is crucial to warrant transmission to new hosts^68^. On these grounds, we tested virulence-relevant traits of the bile-resistant mutants under study by performing reductionist assays *in vitro* (Fig. 2). To our surprise, most mutants characterized in this study (7 out of 10) turned out to be affected in one or more virulence-related traits. The ability of such mutants to thrive inside the gall bladder may thus be accompanied by deleterious consequences in other environments (and the list may fall short to describe other potential long term defects as out-of-host challenges were not tested). Stress-induced mutation in the gall bladder may thus provide genotypic diversity to survive in a harsh niche. However, increased mutation rates may also lead the pathogen towards a dead end in the pursuit of short-term adaptation^9^.

Inefficient colonization of the gall bladder by RpoS^−^ mutants supports the view that non mutational adaptation involves upregulation of the general stress response, a phenomenon previously documented *in vitro*^18^. Additional and/or alternative physiological adjustments may further contribute to survival of planktonic cells in the bile-laden gall bladder lumen^15^. Whatever the molecular mechanisms involved, the frequent occurrence of non mutational adaptation suggests that it may have selective value, and one potential advantage may be avoidance of payoffs associated to mutation.

## Methods

### Bacterial strains

Strains of *Salmonella enterica* serovar Typhimurium (often abbreviated as *S*. *enterica*) derive from the mouse-virulent, streptomycin resistant strain SL1344^69^. Strain SV4898 (*trg::*MudJ Km^R^) was used as wild type during assays of virulence in mice and of proliferation and survival in macrophages. The mutation *trg::*MudJ Km^R^ is neutral for virulence^70^. SV5561 is a RpoS^−^ mutant, and the mutation is null^18^.

### Culture media and growth conditions

Lysogeny broth (LB) was used as standard liquid medium^71^. LB plates contained agar at 15 g/l and streptomycin at 0.2 g/l as final concentrations. To assay *S*. *enterica* bile resistance levels, sodium choleate (ox bile extract, Sigma Aldrich) plates were prepared with stocks of LB agar containing 12, 14, 16, 18 and 20% ox bile extract. In most experiments, cultures were grown at 37 °C with shaking at 250 rpm in a New Brunswick Innova 3100 waterbath. For oral infection of mice, bacterial cultures were grown overnight in LB at 37 °C without shaking. For invasion assays, bacteria were grown overnight in LB broth with 0.3 M NaCl at 37 °C without shaking. For survival/proliferation assays, bacteria were grown 20–24 h in LB broth at 37 °C with shaking as described in Segura *et al*.^70^. Recovery of *Salmonella* from feces was performed as described elsewhere^25^.

### Growth curves

Overnight cultures of the wild type strain and of bile-resistant mutants were diluted 1:25 in LB and incubated at 37 °C with shaking at 250 rpm for 1 h. Aliquots containing around 3 × 10^2^ colony-forming-units (CFU) were transferred to U-bottom 96-well polypropylene microtiter plates (Greiner Bio One) containing either LB or LB with 5% sodium deoxycholate in a final volume of 0.2 ml. The plates were incubated at 37 °C with shaking on an automated microplate reader (Synergy HTX Multi-Mode Reader, Biotek) and the absorbance at 600 nm for each well was measured every 30 min. The duration of each assay was 20 h. The assays were performed in triplicate.

### Isolation and characterization of isolates from mice gall bladders

Eight-week-old female mice belonging to strains BALB/c and 129S2/SvPasCrl, (Charles River Laboratories, Santa Perpetua de Mogoda, Spain), were inoculated with appropriate *S*. *enterica* strains. Information on BALB/c and 129S2/SvPasCrl mice strains can be found at the Mouse Genome Informatics page, http://www.informatics.jax.org/. Bacterial cultures were previously grown overnight at 37 °C in LB without shaking. Oral inoculation was performed by feeding the mice with 25 μl of NaCl 0.9% containing 0.1% lactose and 10^8^ bacterial colony-forming units (CFU). Bacterial cells were recovered from the gall bladder of BALB/c mice 5–10 days after oral infection. Recovery from the gall bladder of 129S2/SvPasCrl mice was performed 70–80 days after the infection. Gall bladder extracts were plated on LB + streptomycin and grown overnight at 37 °C. The following day, the plates were replicated onto LB agar that contained increasing concentrations of ox bile (12, 14, 16, 18, and 20%), and incubated overnight at 37 °C. Bile-resistant mutants were isolated and purified, and the stability of their bile-resistant phenotype was confirmed by determining the MIC of DOC in 96-well plates. Isolates from the gall bladders of mice co-infected with RpoS^+^ and RpoS^−^ strains were recovered by plating identical amounts of gall bladder homogenate on LB kanamycin and LB ampicillin. After 24 incubation, colonies were replica-plated on LB agar containing 12–16% ox bile.

### Determination of minimal inhibitory concentrations (MICs)

Exponential cultures in LB broth were prepared, and samples containing around 3 × 10² colony-forming-units (CFU) were transferred to polypropylene microtiter plates (Soria Genlab) containing known amounts of sodium deoxycholate (DOC) (Sigma Aldrich), the archetypal and most abundant bile salt^72^. Growth was visually monitored after 12–36 h. Similar protocols were used for determination of MICs of polymyxin B sulfate salt (Sigma Aldrich) and hydrogen peroxide. The Student’s *t* test was used to determine whether the differences in MIC values were significant.

### Extraction of genomic DNA

For the extraction of genomic DNA, 5 ml of cells grown to late exponential phase were collected and re-suspended in 0.4 ml of lysis buffer (Tris-HCl 50 mM pH 8, EDTA 10 mM, NaCl 100 mM, SDS 0.2%). Four μl of RNAse (10 mg/ml) were added and the mixture was incubated at 37 °C for 30 min. Twenty μl of a preparation of proteinase K (20 mg/ml) was added and the sample was incubated for 2 h at 65 °C. Finally, 3 or 4 extractions were performed with phenol:chloroform-isoamyl alcohol in a 2:1 proportion. One last extraction was performed with chloroform:isoamyl alcohol (24:1). DNA was precipitated at −20 °C by adding 1/10 volume of sodium acetate 3 M and 2.5 volumes of ethanol. After precipitation, genomic DNA was washed with 70% ethanol and re-suspended in 20 μl of TER buffer (Tris-HCl 100 mM pH 7.5, EDTA 1 mM pH 8, RNAse 20 μg/ml).

### Whole genome DNA sequencing and analysis

Bacterial genome sequencing was performed at the DNA Sequencing Service of the University of Seville (Servicio General de Biología, CITIUS, Universidad de Sevilla, Spain). Sample preparation included gDNA isolation by phenolic extraction and ethanol precipitation. Genome sequencing employed Roche 454 FLX + technology on a GS FLX titanium system. Emulsion PCR and 454 pyrosequencing were performed and 1,031,375 reads with an average read length of 810 base pairs, totaling 1000 Mb. Whole genome sequences were analyzed using the Burrow-Wheeler Alignment tool (BWA), specifically the BWA-MEM algorithm^29,30^. As a reference, the genome sequence of the laboratory stock of *S*. *enterica* SL1344 was also analyzed. Genomes were submitted to NCBI GenBank and are available as SUB3834111 “*Salmonella typhimurium* isolates from Balb/c mice gall bladders (Urdaneta & Casadesus)”. PCR amplification of regions harboring mutations was performed using the primers listed in Table S1. Chromosomal DNA samples obtained by PCR were sequenced by Stab Vida (Caparica, Portugal).

### Eukaryotic cell lines

HeLa cells (ATCC CCL2) and macrophages (RAW264.7) were used as models of nonphagocytic and phagocytic cells, respectively. Cells were routinely cultured at 37 °C with 5% CO_2_ in DMEM medium (Biowest) containing 10% fetal calf serum (Biowest), 4 mM L-glutamine (Biowest) and 1X penicillin-streptomycin solution (Biowest).

### Invasion assays

Invasion assays followed a standard protocol^70^ with slight modifications. HeLa cells resuspended in DMEM were seeded and incubated 24 h before infection in 24 well plates (Thermo Scientific, Denmark) at a concentration of 1 × 10^5^ cells/well. Bacteria were grown overnight (14–16 h) under invasive conditions: LB broth with 0.3 M NaCl at 37 °C without shaking. Bacteria diluted in DMEM were added to reach a MOI of 50:1 bacteria/HeLa cell. Thirty minutes after infection, the cells were washed twice with PBS and incubated in fresh DMEM containing 100 µg/ml gentamicin. Ninety minutes later cells were washed twice with PBS. Numbers of viable intracellular bacteria were obtained after lysis of infected cells with 1% Triton 100-X (prepared in PBS) and plating on LB agar plates. Infections were carried out in triplicate. Invasion rates were determined as the ratio between viable intracellular bacteria and viable bacteria added to infect the HeLa cells. The Student’s *t* test was used to determine whether the differences in invasion rates were significant.

### Survival within macrophages

Survival/proliferation assessment, competitive index (CI) assays with *S*. *enterica* strains were performed as described elsewhere^70^. RAW264.7 macrophages were incubated on 24-well plates (Thermo Scientific, Denmark) 24 h before infection using a concentration of 1 × 10^5^ cells/well. Bacteria were grown for 20–24 h in LB broth at 37 °C with shaking. A 1:1 mixture of the bacterial strains was prepared in DMEM without antibiotics, and added to cultured macrophages to reach a multiplicity of infection (MOI) of 50:1 bacteria/macrophage. Fifteen minutes after infection, cells were washed twice with PBS and incubated in fresh DMEM containing 100 µg/ml gentamicin. Sixty minutes later the concentration of gentamicin was lowered to 16 µg/ml. Numbers of viable intracellular bacteria were calculated by plating on LB X-Gal agar plates after cell lysis with 1% Triton X-100 at two time points: 1 h 15 min after infection and 24 h after infection. Infections were carried out in triplicate. The competitive index in proliferation (CIP) was calculated as described elsewhere^70^. The Student’s *t* test was used to determine whether the differences in competitive indexes were significant.

### Survival in mouse serum

Ten μL of bacterial suspensions containing 1 × 10^4^ bacteria/ml were incubated at 37 °C in 150 μL of either mouse serum (Sigma) with MgCl_2_ 1.3 mM or PBS with MgCl_2_ 1.3 mM. After 2 h incubation, 100 μL aliquots were plated on LB agar. Survival rates were determined as the ratio between viable bacteria treated with mouse serum and viable bacteria incubated in PBS. The Student’s t test was used to determine whether the differences in survival rates were significant.

### Virulence assays in mice

BALB/c mice (Charles River Laboratories, Santa Perpetua de Mogoda, Spain) were inoculated with a 1:1 mixture of strains SV5561 and SV4898. Bacterial cultures were previously grown overnight at 37 °C in LB without shaking. Oral inoculation was performed by feeding the mice with 25 µl of PBS containing 0.1% lactose and 10^8^ bacterial CFU. Bacteria were recovered from the gall bladder 7 days post-infection. A competitive index (CI) was calculated as described by Beuzón and Holden^73^.

### Ethics statement

Animal research adhered to the principles mandatory in the European Union, as established in the Legislative Act 86/609 CEE (November 24, 1986) and followed the specific protocols established by the Royal Decree 1201/2005 of the Government of Spain (October 10, 2005). The protocols employed in the study were reviewed by the Comité Ético de Experimentación de la Universidad de Sevilla, and were approved on January 16, 2010 (permit number 59-A-2010).

## Supplementary information

Supplementary information
