# Supplementary material for: Mutational and non mutational adaptation of Salmonella enterica to the gall bladder
Source: Sci Rep. 2019 Mar 26;9:5203. doi: 10.1038/s41598-019-41600-8 (PMC6435676; doi:10.1038/s41598-019-41600-8)
Supplement: Supplementary file 1 — Supplementary information [file 41598_2019_41600_MOESM1_ESM.pdf]

Mutational and non mutational adaptation of *Salmonella enterica* to the gall bladder

Verónica Urdaneta, Sara B. Hernández and Josep Casadesús

SUPPLEMENTARY INFORMATION

Table S1. Oligonucleotides used in this study

Oligonucleotide name	Sequence (5' → 3')
rlpB-E1	CAATGGTGGAAAACACCACG
rlpB-E2	GGATCGAGAGTAAAAGCGTG
pbgA-E1	GAAAAACATAAGGCGCCGAC
pbgA -E2	AGCCGTAACAGGCTGGTTC
dipZ-E1	ACATCGCTGTACTACTGG
dipZ-E2	TGGCCAGAAACGCTGTAATG
ftsQ-E1	CAGGCATGGAAAGCTTTAGG
ftsQ-E2	GAACGCATTTCACCACTGA
STnc400STM3845-E1	GAGATGCTTCAGTCTTGG
STnc400STM3845-E2	GGATGAGCAATGGTTCTC
dam-A	CAATTGCCTGTGAGTGTCG
dam-B	CAGCCGACAGAATTG
ftsK-E1	ACGTAGTGATGGAAGAGG
ftsK-E2(internal)	ATTGCGGATGCGGCTGATAC
STM1268-E1	TGCTGCTGCGATTGCAATAG
STM1268-E2	ACATGCGTTCATTGAGCG
ygcF-E1	GTAATGGATGAGTCACAGACG
ygcF-E2	GAAGCTCTGGGCGAAAAAGC
3216501-E1	CCTTTCTGGATATACAACG
3217010-E2	GAAGCTCTGGGCGAAAAAGC
yhbG-E1	TGACCGGCAACGCTTATCTG
yhbG-E2	ATCGGTTTGCTCAAGCAG
2728222-E1	AGAGCAGCCCTATTGGAGT
2729110-E2	CCTCCTAAGGAAAACCACAAG
yifK-E1	TCTGATGACTTGAGCAGC
yifK-E2	AACCCCCACGTCCGTAAGGAC
Glmz-E2	GGACGCGTATGATTATGCCTG
